# Printing the Future—Updates in 3D Printing for Surgical Applications

**DOI:** 10.5041/RMMJ.10343

**Published:** 2018-07-30

**Authors:** Dekel Shilo, Omri Emodi, Ori Blanc, Dani Noy, Adi Rachmiel

**Affiliations:** 1 Department of Oral and Maxillofacial Surgery, Rambam Health Care Campus, Haifa, Israel; 2 Bruce Rappaport Faculty of Medicine, Technion–Israel Institute of Technology, Haifa, Israel

**Keywords:** Computer-assisted, craniofacial, implants, reconstructive surgical procedures, stereolithography, three-dimensional printing

## Abstract

Three-dimensional (3D) printing is based on additive technology in which layers of materials are gradually placed to create 3D objects. The world of 3D printing is a rapidly evolving field in the medical industry as well as in most sectors of our lives. In this report we present current technological possibilities for 3D printing in the surgical field. There are different 3D printing modalities and much confusion among clinicians regarding the differences between them. Three-dimensional printing technologies can be classified based on the basic material used: solid, liquid, and powder. We describe the main printing methods from each modality and present their advantages while focusing on their applications in different fields of surgery, starting from 3D printing of models for preoperative planning up to patient-specific implants (PSI). We present the workflow of 3D printing for the different applications and our experience in 3D printing surgical guides as well as PSI. We include examples of 3D planning as well as clinical and radiological imaging of cases. Three-dimensional printing of models for preoperative planning enhances the 3D perception of the planned operation and allows for preadaptation of surgical instruments, thus shortening operation duration and improving precision. Three-dimensional printed PSI allow for accurate reconstruction of anatomic relations as well as efficiently restoring function. The application of PSI is expanding rapidly, and we will see many more innovative treatment modalities in the near future based on this technology.

## WHAT IS 3D PRINTING?

Three-dimensional (3D) printing is based on additive technology in which layers of materials are gradually placed to create 3D objects. In this technology objects are created by controlled addition of material, rather than subtraction.

The technology, which started as a method used for rapid prototyping, was first patented by Charles Hull in 1984. Hull described his invention as: “A system for generating three-dimensional objects by creating a cross-sectional pattern of the object to be formed at a selected surface of a fluid medium capable of altering its physical state in response to appropriate synergistic stimulation.”^1^ Hull is considered the inventor of the stereolithography (SLA) method, which is based on solidifying layers of photopolymer resin.

Historically, 3D printing was developed for industrial and engineering use. Early on, it focused on rapid prototyping, generating physical models of a component or system for visualization purposes. The technology developed to allow for rapid manufacturing of complete complex products.^2^

**Figure 1:**
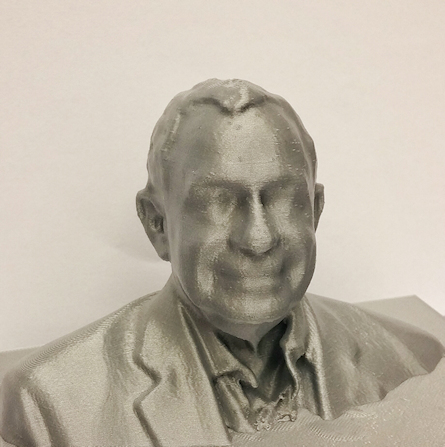
A 3D Printed Replica of the Director of Rambam Health Care Campus This was printed using a fused deposition modeling printer.

The world of 3D printing is a rapidly evolving field in the medical industry as well as in most sectors of our lives and lately even assimilating into many households, which acquire the technology due to the cost-reduced options in the market and the possibilities hidden inside it for almost any of us (Figure 1). Nowadays almost everyone knows the technology exists.

Personalized medicine can be defined as selecting appropriate therapies based on a patient’s genetic content or other molecular or cellular analysis. This approach is rapidly developing in cancer treatment, for example.

When talking about 3D printing and personalized medicine, we use 3D imaging for planning and creating solutions based on the physical structure of a specific tissue.

## TECHNOLOGIES OF 3D PRINTING

There are different classification methods for the different printing modalities. One way to classify the printing methods relies on the basic material used: solid, liquid, and powder.

There is much confusion among clinicians regarding the differences between different technologies for 3D printing. For example, most do not know the difference between selective laser sintering, direct metal laser sintering, and selective laser melting, all of which are members of the powder-based methods.

We will describe the main methods from each group, emphasizing the differences, advantages, and disadvantages.^3^,^4^

- *Liquid based:* This category contains the oldest form of rapid prototyping, SLA. Stereolithography is based on an ultraviolet laser which polymerizes light-curable resin, solidifying specific areas in layers on a mobile platform which descends as the process progresses into a container of resin, thus successive layers of resin are cured on top of each other.
- *Solid based:* A widespread example of a solid-based printing modality is the fused deposition modeling (FDM) which is based on continuous deposition of material. In this method layers are created by the deposition of a heat-softened thermoplastic material. This method is used in most economical consumer printers.
- *Powder based:* Selective laser sintering (SLS) is based on a powder bed in which a high-powered laser heats the powder particles to a point that the powder can fuse on the molecular level, forming a solid layer. The tray then descends and a new layer is fused on top of the previous one. Selective laser melting (SLM) is a bit different. When the laser heats up the material powder to just below the melting point it is considered as SLS, and if it heats to just above the melting point it is considered as SLM. The differences are mainly in the porosity of the material; in SLS there is some porosity, which does not exist in SLM. On the other hand, SLM requires a purer substance, while in SLS alloys may be used. The term direct metal laser sintering refers to the same process as SLS but includes only metal alloys, while SLS includes a variety of materials. Another technology with rising popularity is electron beam melting (EBM). It is similar to SLM, and the difference is that EBM uses an electron beam instead of laser.

There are other technologies in the liquid, solid, and powder-based groups; however, it is beyond the scope of this paper to further elaborate on each of them.

It is important to define what the objective of the print is. For printing models to allow preadaptation of fixation plates, presenting findings, or preoperative planning of the surgery one can use SLA or FDM. When printing implants, SLS is usually the way to go. Common sterilization techniques for objects used intraoperatively or for implantation include high-temperature, chemical, or radiation sterilization.^5^ It is important to remember that many of the materials used to create surgical guides are heat-sensitive due to their low melting point and thus require special sterilization protocols such as ethylene oxide.^6^,^7^ However, metal powder bed fusion results in implants which can withstand autoclaving.

An important note is that in contrast to SLA and FDM, which most often require support structures to print overhangs in objects, SLS does not need supports because the surrounding powder supports the unconnected parts; this allows for printing of previously impossible geometries. In SLA and FDM supports are essential because of the time required for the thermoplastic material to harden and thus for the bonding of the layers.^8^–^10^

## CLINICAL APPLICATIONS IN SURGERY

Most of the surgical departments nowadays have tried using 3D printing in one way or another, starting from visual-tactile aids for preplanning surgery and up to complete virtual planning of the surgery and customized surgical guides as well as patient-specific implants (PSI) which stay in the living body.

Most of the applications of 3D printing in surgery focused on these three categories: surgical 3D models, surgical guides, and implants. While models and guides can be printed using SLA and FDM, implants are usually printed using SLS, SLM, or EBM. There are many reports in the literature describing the use of all three categories in surgery.

Printing life-size anatomic models can benefit in several aspects, including education of young surgeons on models allowing for tactile and 3D inspection of the tissues. The models can also be further used for performing mock surgeries thus improving the prediction of the outcomes. These models may also be used for presurgical adaptation of instrumentation, thus reducing the operation time and achieving superior compatibility.

**Figure 2:**
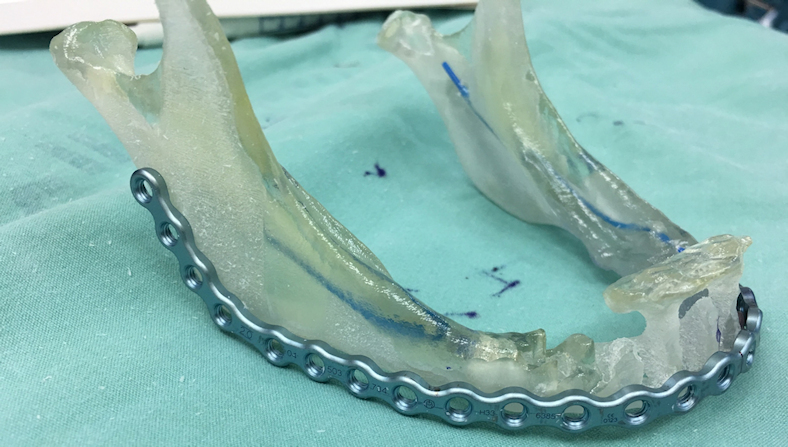
3D Printed Model of a Lower Jaw This patient was planned for anterior resection of the mandible due to osteomyelitis, and thus a reconstruction titanium plate was used for fixation of the remaining bony fragments. A 3D model was printed allowing for pre-bending of the reconstruction plate prior to the operation, thus reducing operation duration and allowing for easy and accurate adaptation of the plate following the resection.

**Figure 3:**
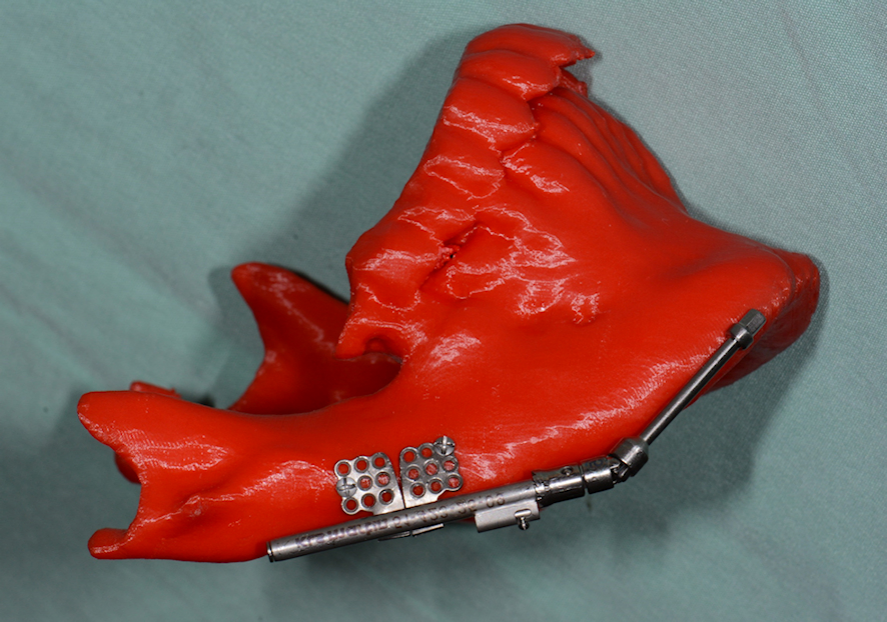
An Example of Using a 3D Printed Lower Jaw Model Preoperatively for Distraction Osteogenesis Device Selection In this process bone elongation is performed which results in new bone and soft tissue augmentation.

Three-dimensional printed models were shown to be superior in preoperative planning compared to 3D images.^11^ These applications were used in many fields such as vascular surgery for printing aortic models, in endovascular aneurysm repair to select the proper device,^12^,^13^ in cardiac surgery for presurgical planning of tumor resections and repair of congenital defects,^14^,^15^ in neurosurgery for navigation training,^16^ and in orthopedic surgery for planning of tumor resection and treatment of trauma injuries.^17^,^18^ We used 3D printed models in cranio-maxillofacial surgery for pre-bending of reconstruction titanium plates on a 3D model of the skull prior to resections, thus allowing us to restore the correct position of the remaining bones accurately while reducing the operation length (Figure 2). We also 3D printed models for preoperative distraction osteogenesis device selection (Figure 3).

As virtual planning gains more popularity, especially with virtual reality developing rapidly and PSI becoming a standard of care, printing 3D models will lose its current popularity, yet an interesting application gaining momentum is surgical guides. These allow for accurate surgical resections or osteotomies based on preoperative imaging. Using these guides with the intention of inserting PSI is essential as accuracy is of very high importance, especially when metal implants are used which are extremely difficult to amend during surgery.

**Figure 4:**
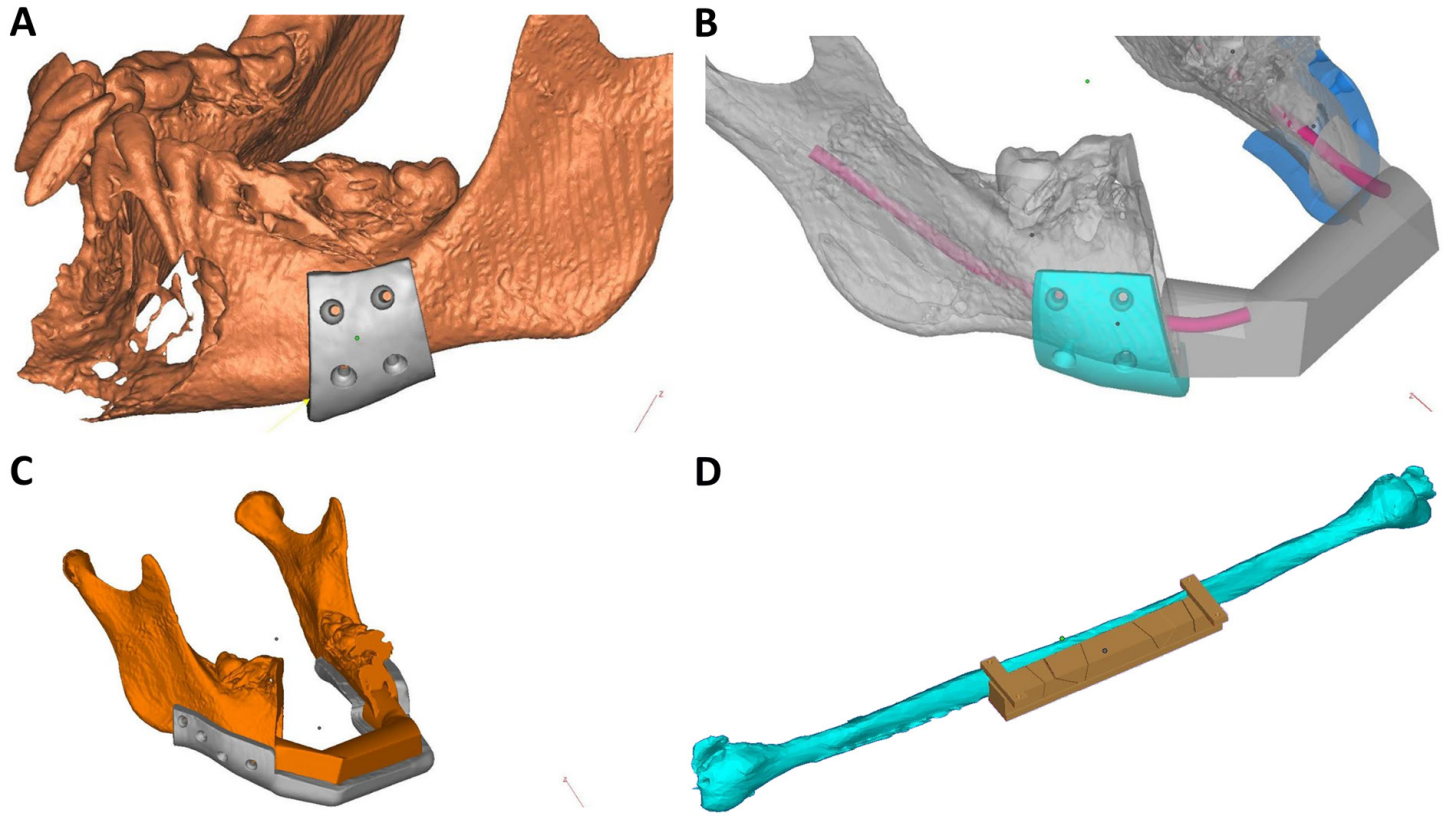
Surgical Guides A patient undergoing anterior mandibular resection due to an aggressive tumor is presented. A fibula free flap was chosen for the reconstruction. **A:** Surgical cutting guides were designed allowing for accurate resection. **B:** Reconstruction using a fibula free flap was planned. **C:** An intraoperative guide for accurate placement of the harvested flap was designed. **D:** A respective cutting guide for the fibula is designed allowing for a perfect flap harvest including supplemental positioning of the osteotomies performed on the fibula bone to create the final three-piece bone graft which will reconstruct the mandible.

**Figure 5:**
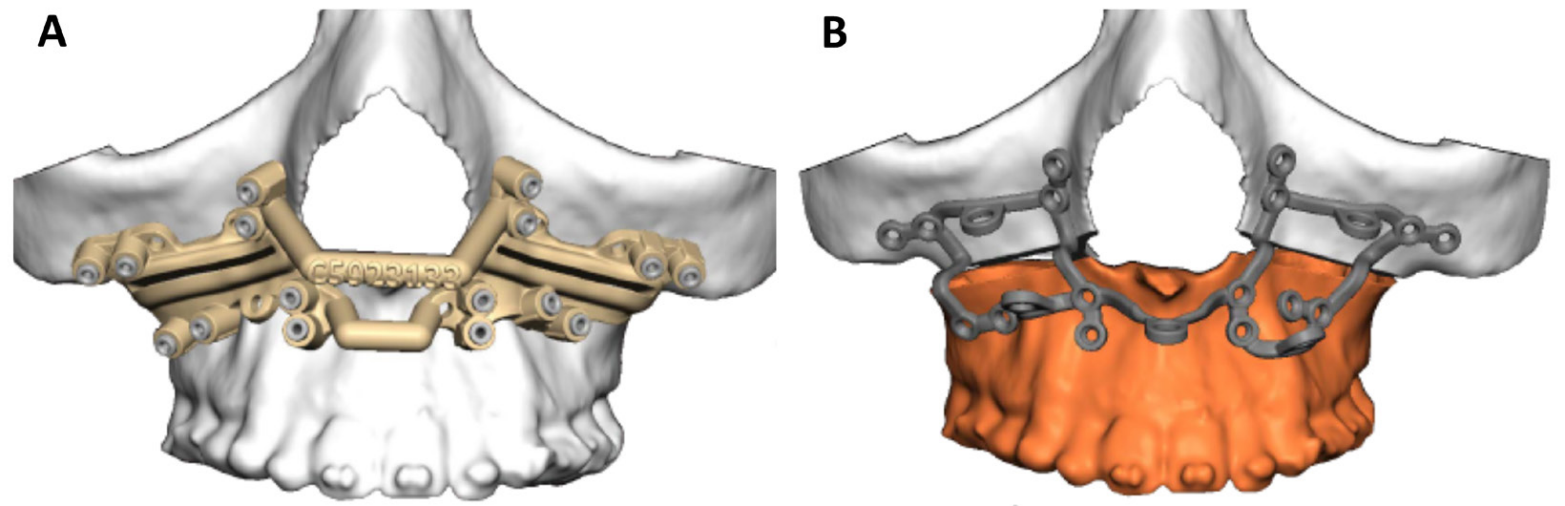
Cutting Guides in Orthognathic Surgery When performing orthognathic surgery to correct the position of the upper and lower jaws one can use a wafer intraoperatively to position the jaws and bend plates intraoperatively, or use 3D printed cutting guides **(A)** to perform accurate osteotomies in 3D preplanned waferless operations prior to placement of patient-specific fixating plates **(B)**.

**Figure 6:**
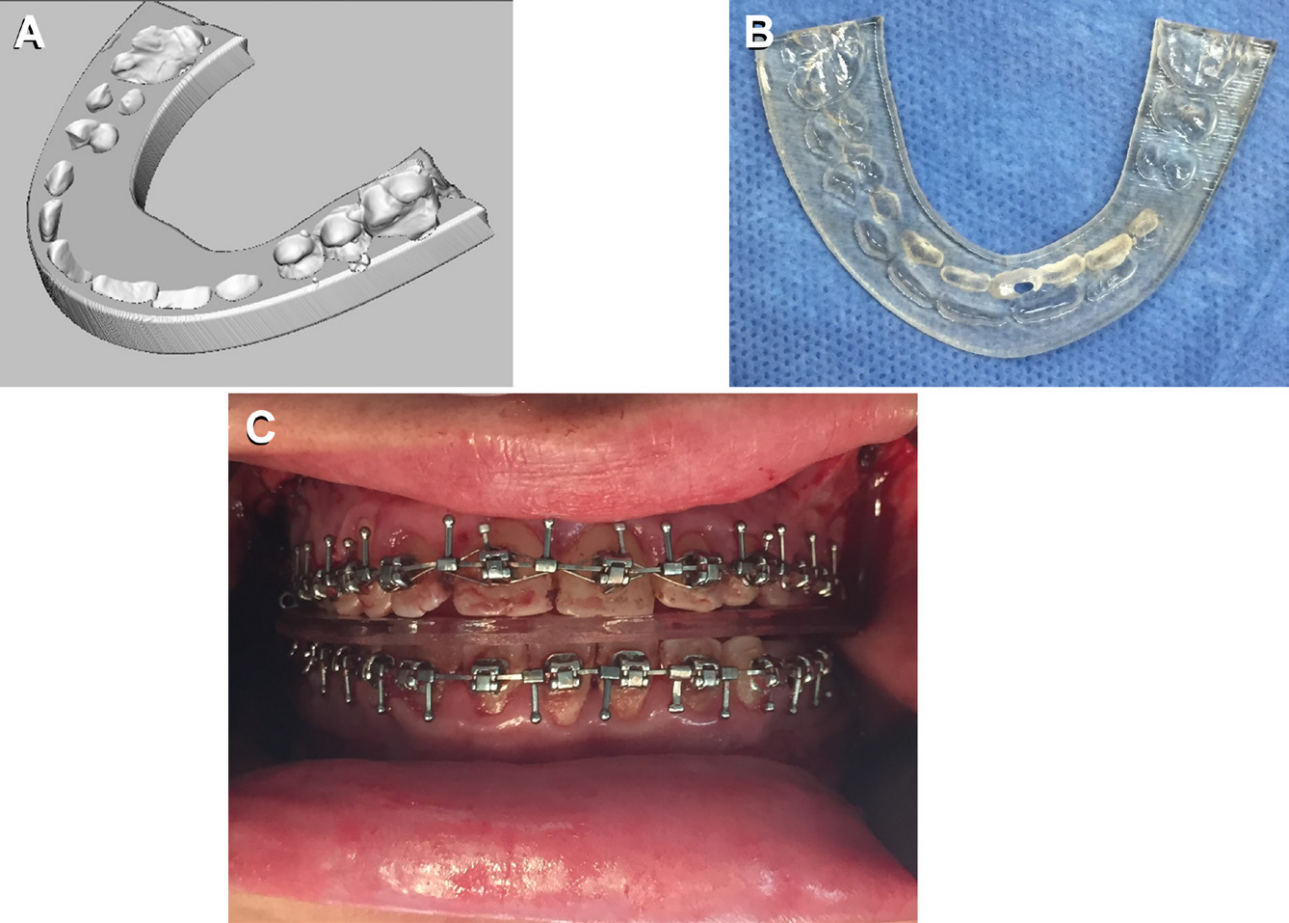
In Orthognathic Surgeries One Can Use Intraoperative 3D Planned and Printed Dental Splints for Accurate Repositioning of the Jaws/Midface **A:** 3D planning of the splint. **B:** The printed splint. **C:** Intraoperative positioning of the jaws according to the splint.

One of the most described uses of surgical guides in cranio-maxillofacial surgery is their application for bone resections and free flap reconstruction using a fibula free flap, for example (Figure 4). We have used 3D planning and intraoperative guides for accurate rib grafting and fixation in mandibular ramus deficiencies.^19^ Another popular use is for orthognathic surgery. Orthognathic surgery is a corrective surgery, aiming to restore the proper anatomic and functional relationship in patients with dentofacial skeletal anomalies. The classic approach involved using an articulator and dental casts to transfer the skeletal relations, mock surgery on the casts based on our measurements, and acrylic wafers as guides in the operation room for repositioning of the jaws. Nowadays, 3D preplanned waferless operations can be used for performing accurate osteotomies and perfect positioning of the unaligned jaw. Three-dimensional printing of cutting guides for the osteotomies and 3D printed patient-specific fixating plates for accurate final positioning of the jaws, based solely on the 3D preoperative planning, greatly reduce the incorporation of human errors (Figure 5).^20^ Intraoperative 3D printed dental splints for accurate repositioning of the jaws/midface based on 3D preoperative planning can also be prepared in cases where patient-specific fixating plates are not an option (Figure 6). In orthopedics cutting guides were used as drill guides and as guides for harvesting cartilage,^21^,^22^ as well as for resections.^23^

The most recent and advanced use of 3D printing in the surgical field is PSI. Patient-specific implants are planned based on accurate 3D imaging and thus result in perfectly fitting implants used to restore proper anatomy, relation, and function. In cranio-maxillofacial surgery PSI are becoming extremely prevalent with numerous companies offering various implants from different materials intended both for function and restoring anatomy and symmetry. We used titanium implants for load-bearing reconstruction following mandibular resections and avulsion injuries combined with autogenous bone grafts,^24^ as well as customized PSI integrated with dental implants for future dental arch and occlusion restoration.^25^ In addition, we used polyether ether ketone (PEEK) implants for restoration of deficiencies in the zygomatico-orbital complex and mandibular angles, both as late repair of trauma injuries and in syndromic patients, as well as PEEK and titanium for restoration of orbital wall defects. The PEEK implants allow for minor adjustments in the operating theater, while titanium implants are not adjustable but demonstrate increased strength.

**Figure 7:**
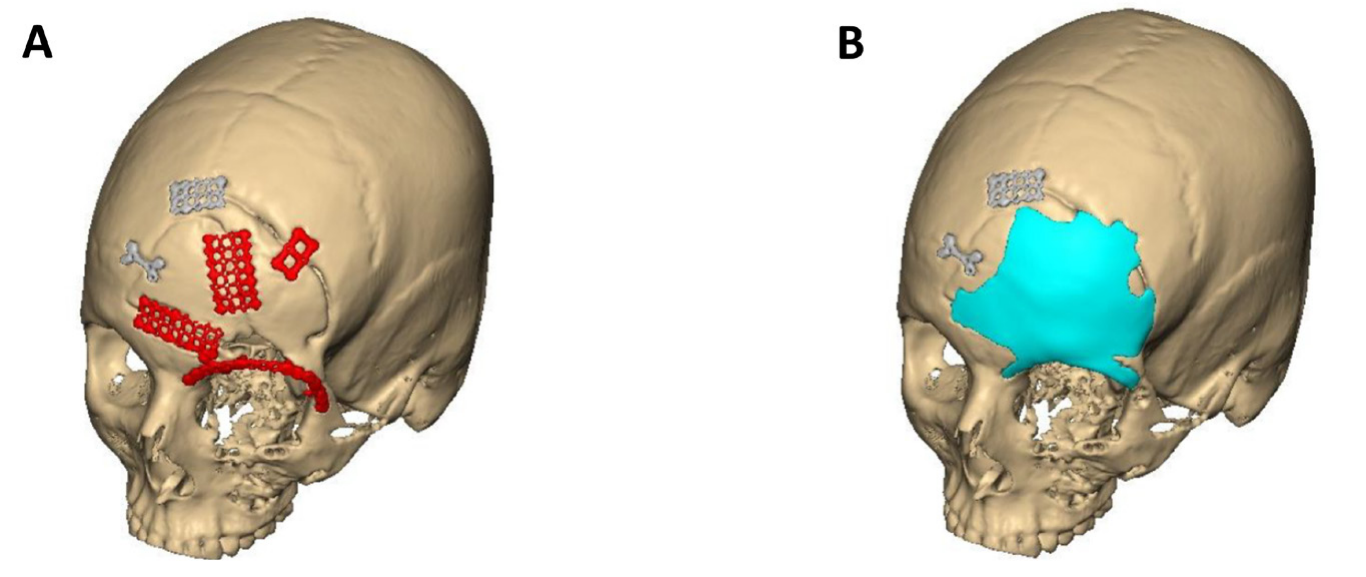
Frontal Bone Defect An alloplastic PEEK implant was designed to reconstruct the bony defect in the left frontal bone, including the superior orbital rim. **A:** Following reduction and fixation of traumatic injury; red fixation plates will be removed. **B:** Planning the PEEK implant to reconstruct the defect.

Patient-specific implants were also used in orthopedics for bony reconstruction following tumor resections,^6^ for printing customized external fixators used in treating fractures,^26^ and in cervical spine reconstruction.^27^ Neurosurgery is another field embracing PSI technology, mostly in cranioplasty for reconstructing skull defects (Figure 7).^28^,^29^ In thoracic surgery implants were used to reconstruct the chest wall,^30^ and in ophthalmology for ocular prosthesis.^31^

## WORKFLOW

**Figure 8:**
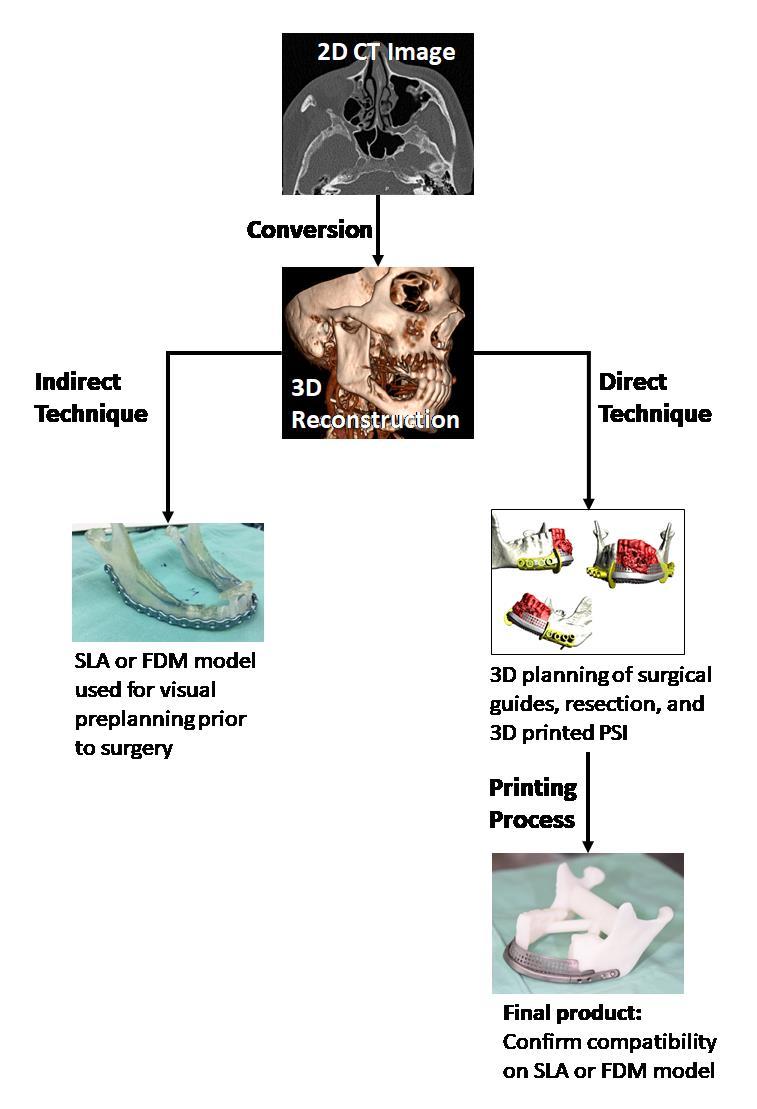
The Workflow of Using 3D Printing in Surgical Applications Is Presented First a 3D reconstruction of 2D slices is performed. In the indirect technique a model can be printed for further preoperative planning. In the direct technique computer-assisted design programs are used to design surgical guides and patient-specific implants. These aids can later be inspected using a 3D print of the 3D reconstructed tissue as in the indirect technique.

The workflow of using 3D printing in surgical applications is described in Figure 8. The process begins with 2D sections of different imaging utilities such as computed tomography or magnetic resonance imaging. Image acquisition is critical because the final accuracy of the object depends on the quality of the acquisition. Image sections should be reconstructed with isotropic voxels of 1 mm or less.^32^ Thin sections will require much processing, and thick sections result in a less accurate result. Cardiac models appear accurate when acquiring 0.5 mm sections.^33^ Thin objects such as the orbital floor requires thinner sections.^34^

**Figure 9:**
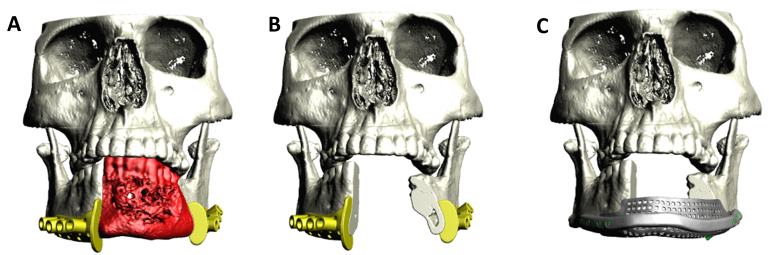
Three-dimensional Planning of Surgical Guides and Patient-specific Implants **A:** Surgical cutting guides are designed to accurately position the osteotomies for a segmental resection of the anterior mandible. Bone painted in red represents the resected bone. Notice the included guiding paths for drilling holes indicating future screw placement in the patient-specific plate. **B:** Following resection. **C:** The planned patient-specific implant including a meshed crib allowing placement of autogenous bone graft for future dental implant insertion.

**Figure 10:**
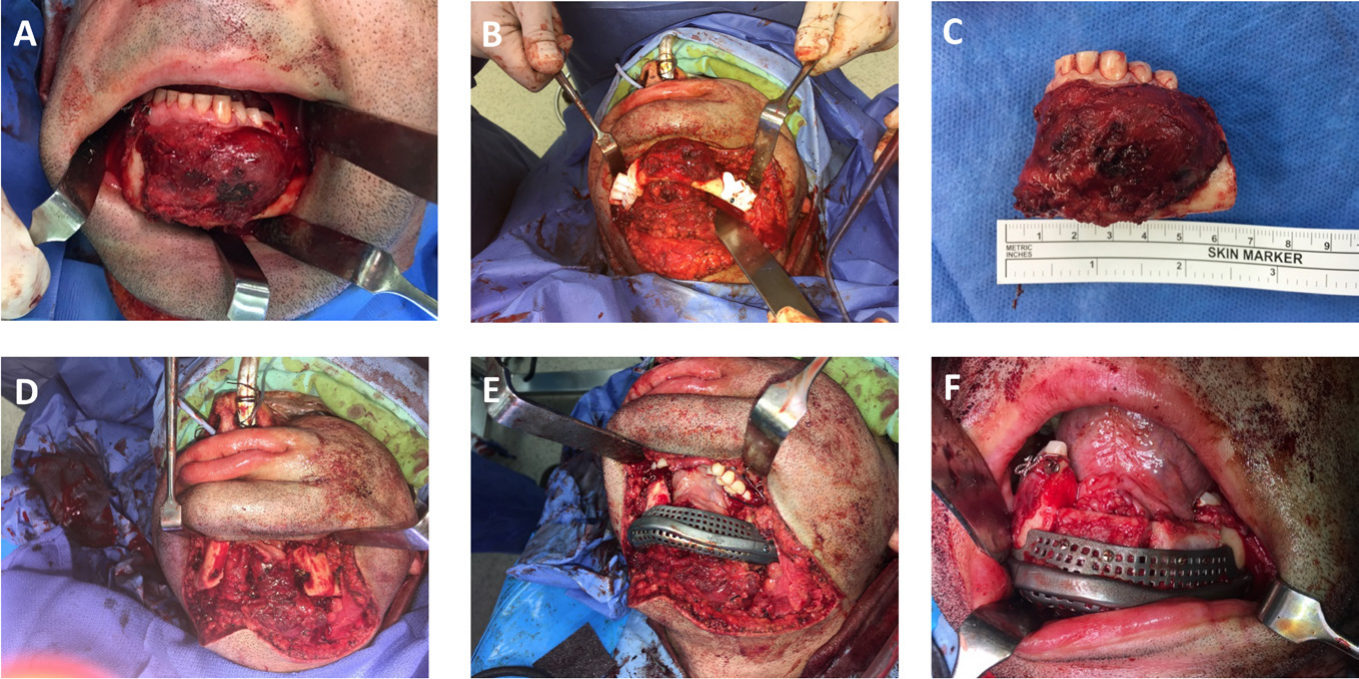
The Operation of the Patient in Figure 9 Is Presented **A:** Tumor exposure. **B:** Cutting guides are placed**. C and D:** Following resection**. E:** Insertion of the patient-specific implants without the need for external fixator as the bony relations are re-established by the drilled holes performed earlier using the cutting guides. **F:** Placement of the bone graft in the meshed crib.

**Figure 11:**
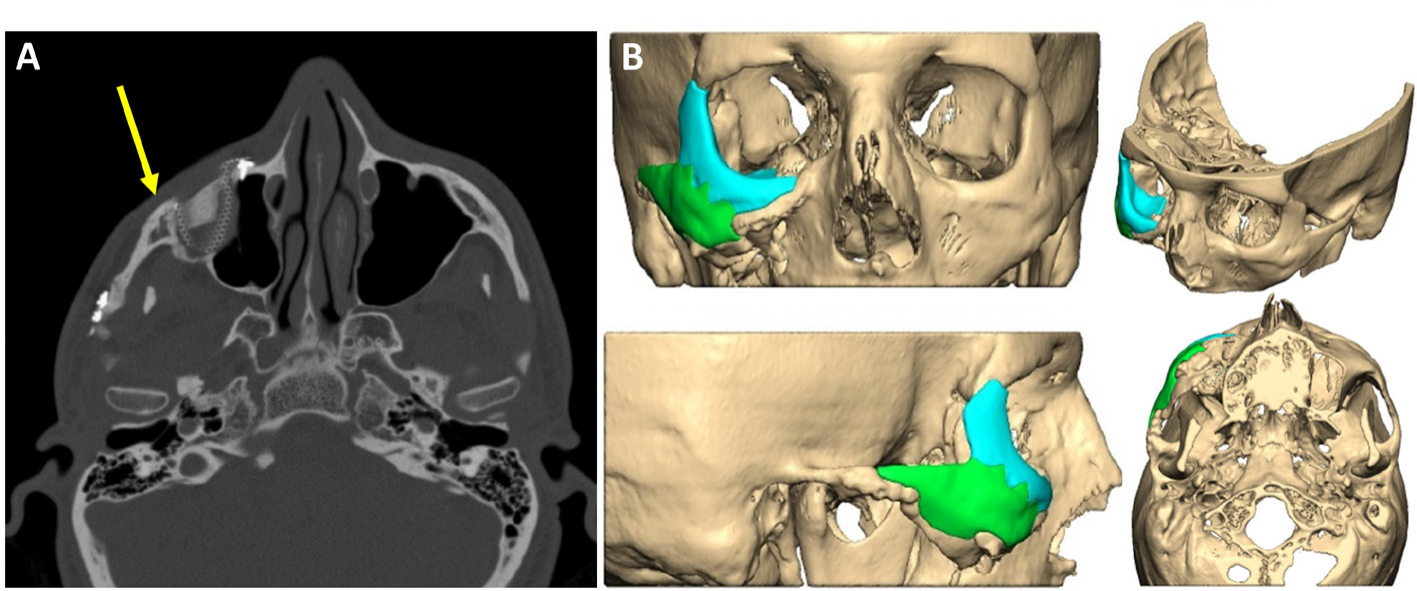
Patient-specific Implant for Reconstructing the Zygomatico-orbital Complex **A:** Axial CT reconstruction showing a patient suffering from improper reduction of fractures in the zygomatico-orbital complex. Yellow arrow shows the lacking projection of the malar eminence on the right side. **B:** A patient-specific implant made of polyether ether ketone (PEEK) was designed.

**Figure 12:**
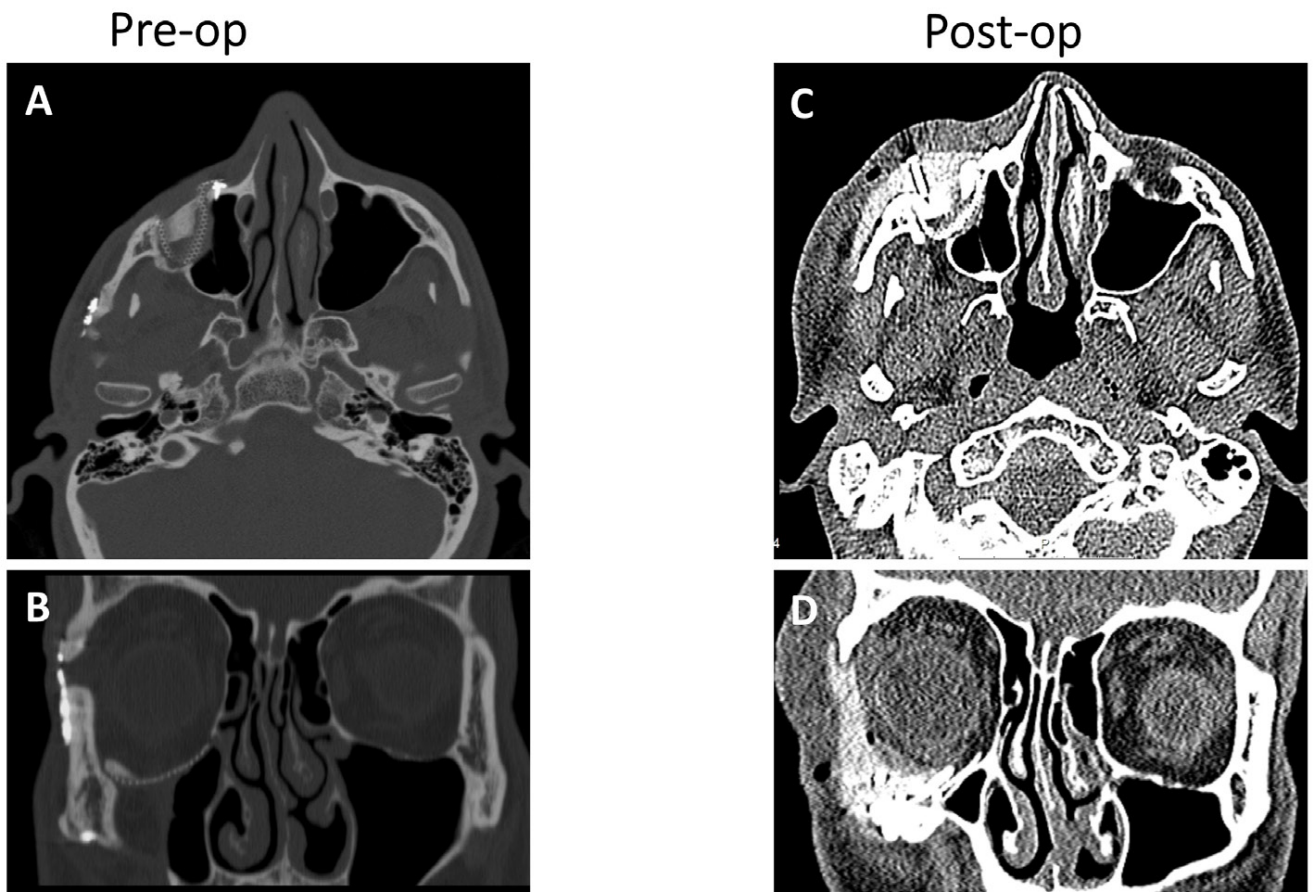
Pre- and Postoperative Computed Tomography Imaging of the patient in Figure 11. **A and B:** Axial and coronal computed tomography reconstructions, respectively, showing the deficiency in the right malar eminence and the increased orbital volume. **C and D:** Axial and coronal computed tomography reconstructions, respectively, showing proper orbital volume and projection of the malar eminence. Notice the need for soft tissue window computed tomography reconstruction as the polyether ether ketone (PEEK) implant does not have the same radiopacity as bone.

The next stage involves 3D rendering of the 2D slices. This process creates a standard tessellation language (STL) file which can now be processed (segmentation and surface preparation) and printed as a model using discussed methods such as SLA or FDM. For some, this is as far as they proceed with 3D printing for surgical purposes, and they use the printed model for preplanning of the surgery. This technique is termed the indirect technique. Nowadays, technology has evolved, and some surgeons proceed to the next stage which is performed using advanced computer-assisted design software (CAD) for virtually designing the operation. This way the surgeon can preplan the operation and 3D print aiding objects as well as perfectly adapted alloplastic replacement implants. This technique is considered the direct technique. Examples of this kind of planning includes PSI which are perfectly adapted to the remaining tissue and can fixate bony fragments to restore proper contour and facial symmetry (Figures 9–12). Another example of aiding objects are cutting guides used for resections or osteotomies. These guides fit perfectly on the tissue and mark the exact place to perform the osteotomies. They may also include guiding paths for drilling holes indicating future screw placement in the fixating patient-specific plate (Figure 9A, B). These can later guide the surgeon to the right placement of the fixating plate without the need for an external fixation device to maintain proper relations between the remaining fragments following resection (Figures 9–10). The planning is usually performed by an engineer based on the request of the surgeon. This requires a cross-talk between the engineer and the surgeon until a satisfactory result is achieved. The surgical guides and the PSI are 3D printed using the requested materials. When ordering PSI, one can ask for an SLA model to confirm the compatibility of the implant and to “feel” the implant on the simulated remaining tissue. The ordered objects usually arrive sterile and are ready for the operation. During the operation one should make sure to properly expose the target area to allow for a perfect fit of the surgical guides and PSI with no interferences (Figure 10B).

Results may be confirmed via intraoperative computed tomography or postoperative imaging. One should take into consideration that some materials are difficult to assess using radiological imaging (Figure 12).

## THE FUTURE

Planning is performed nowadays by engineers. This is due to several reasons. For one, most of the CAD programs available today were intended for the industrial fields, and thus are not user-friendly for the surgeon who usually lacks appropriate education. Another reason is the need for structural analysis of the implants with respect to biomechanical aspects. This way of planning results in the need for a cross-talk between the engineer and the surgeon, who are often in different countries and speak different languages. With time, planning will be simplified and become more user-friendly, taking into account mechanical issues and implementing rules for virtual planning, making sure the implant will maintain stability under physiological forces. This will shift the planning process to the surgeon, thus saving the time-consuming, costly cross-talk between engineers and surgeons.

Using PSI results in a more precise and durable method for reconstruction, with lower morbidity and shorter operation time. Yet these alloplastic materials have their disadvantages: they are still foreign bodies and are thus prone to infection and oral/cutaneous dehiscence, and the fixating screws can loosen and create an inflammatory reaction.

The future lies in 3D bio-printing of viable cells which will compose the missing bone and soft tissue. The field of bio-printing is extensively investigated, leading to improvement in technologies, materials, and protocols. Although the field is considered to be in its early phases of development, human-scale tissues have already been printed; examples include skin, cartilage, vascular tissue, aortic valve, and kidney.^35^ Technological challenges include the need for increased resolution, speed, and compatibility with biologically relevant materials. Of course, vascularization, which is a great challenge in tissue engineering, is also a major obstacle in bio-printing: proper vascularization of the 3D printed tissue must be achieved for long-term viability.^35^ Until bio-printing becomes a standard, a noteworthy application is 3D printing of bioresorbable implants. An example is creation of a bioresorbable polycaprolactone airway splint that was implanted in a boy suffering from tracheobronchomalacia.^36^

## CONCLUSIONS

Three-dimensional printing of models for preoperative planning enhances the 3D perception of the planned operation, either as a visual-tactile aid or for performing mock surgeries. It allows for preadaptation of surgical instruments such as fixation plates and thus shortens the operation and improves precision.

Three-dimensional printed PSI allows for accurate reconstruction of anatomic relations as well as efficiently restoring function. A PSI spares the need for adaptation in the operating theater, thus resulting in highly resistant implants which can easily withstand physiological forces. The application of PSI is expanding rapidly, and we will see many more innovative treatment modalities in the near future based on this technology.

Patient-specific implants planning begins with the surgeon, continues in the hands of the engineer, returns to the surgeon and so on, while live web meetings may be performed for quicker results. Is this the proper and most efficient method for planning PSI? Probably not. As CAD programs evolve, they will be more user-friendly and will decrease the role of the engineer in the process, perhaps leaving him with only structure integrity verification prior to printing the implant. Bio-printing will be the ultimate tool to reconstruct missing tissues and thus resolving the disadvantages of alloplastic implants. We are far from the day when this method will be part of our toolbox, yet it will completely change how we think and operate when we get there.

## Abbreviations

CAD: computer-assisted design
EBM: electron beam melting
FDM: fused deposition modeling
PEEK: polyether ether ketone
PSI: patient-specific implants
SLA: stereolithography
SLM: selective laser melting
SLS: selective laser sintering.
